# An Association Between Comorbidities and Postsurgical Complications in Adults Who Underwent Esophagectomy

**DOI:** 10.7759/cureus.36395

**Published:** 2023-03-20

**Authors:** Alexandra N Townsend, Alexa Denton, Nishant Gohel, Juan Lozano, Pura Rodriguez de la Vega, Grettel Castro, Rupa Seetharamaiah

**Affiliations:** 1 Department of Medical and Population Health Sciences Research, Herbert Wertheim College of Medicine, Florida International University, Miami, USA; 2 Department of Surgery, Herbert Wertheim College of Medicine, Florida International University, Miami, USA; 3 Department of Surgery, Baptist Hospital of Miami, Miami, USA

**Keywords:** postoperative complication, nsqip, comorbidities, esophageal cancer, esophagectomy

## Abstract

Background

Esophagectomy is the surgical excision of part or all of the esophagus and is associated with both common and serious complications. Various comorbidities, such as diabetes mellitus, smoking, and congestive heart failure (CHF), have been detected in individuals who have undergone esophagectomy. This study investigates the association of baseline characteristics and comorbidities with postoperative complications.

Methods

A retrospective cohort study based on data from the National Surgical Quality Improvement Program database was conducted, evaluating 2,544 patients who underwent esophagectomy between January 2016 and December 2018. Data included baseline characteristics, established comorbidities, and postoperative complications within 30 days of the procedure. Risk-adjusted and unadjusted logistic regressions were used to assess the odds of postoperative complications against baseline characteristics.

Results

The majority of our population were white males (80.8% male; 78.2% white), with a mean age of 63.5 years. More than half of the patients were overweight or obese. A minority of our patients had a smoking history, weight loss, diabetes mellitus, chronic obstructive pulmonary disease (COPD), or CHF. The most frequent postoperative complications were as follows: return to the operating room (15.7%), anastomotic leak (12.9%), pneumonia (12.7%), bleeding/transfusions (11.8%), readmissions (11.4%), and unplanned intubation (10.5%). Adjusted associations for odds of experiencing a postoperative complication were found to be statistically significant for age (odds ratio [OR] 1.02, 95% confidence interval [CI] 1.01-1.03, and *P *< 0.001), operation time (OR 1.002, 95% CI 1.001-1.003, and *P *< 0.001), race (not white) (OR 1.76, 95% CI 1.26-2.47, and *P *= 0.001), BMI (underweight) (OR 2.18, 95% CI 1.36-3.50, and *P *= 0.001), smoking (OR 1.42, 95% CI 1.14-1.76, and *P *= 0.001), and chemotherapy and/or radiation (OR 0.82, 95% CI 0.68-0.99, *P *= 0.038).

Conclusions

Our study found that age, operation time, nonwhite race, underweight BMI, and smoking were independently associated with an increased risk of developing a postoperative complication following esophagectomy. Additionally, neoadjuvant chemotherapy and/or radiation are associated with a decreased risk. Understanding how baseline characteristics and comorbidities can affect rates of postoperative complications may help to adjust care for patients in both pre- and postoperative settings.

## Introduction

Esophageal cancer is one of the deadliest malignancies, with 20,640 new cases and 16,410 deaths reported in 2022 [1]. The five-year survival rate across all stages is roughly 20%, and the highest rate of this condition occurs in men who make up 78% of cases and 81% of deaths [1]. Esophagectomy, the partial or full surgical resection of the esophagus, is the current gold standard in care for patients with esophageal cancer, such as esophageal adenocarcinoma or squamous cell carcinoma [2,3].

Although the gold standard, esophagectomies can lead to various complications that can impact overall patient outcomes and may lead to an increase in mortality. The most common complications, causing nearly two-thirds of mortality following esophagectomy, are pulmonary conditions such as acute respiratory distress syndrome, acute exacerbation of chronic obstructive pulmonary disorder (COPD), or pneumonia [4]. While pulmonary complications are more common, anastomotic leaks following esophagectomy are the most feared [4]. Studies have shown that several patient risk factors are associated with an increased risk of an anastomotic leak such as heart failure, type of procedure, renal insufficiency, and hypertension [5]. Additional notable complications include wound infections, renal failure, urinary tract infections, cardiac complications, sepsis, and death [4,6-8].

Studies have examined the relationship between various specific patient comorbidities and esophagectomy postoperative complications; however, few studies holistically analyze the association between comorbidities and the associated risk of developing a listed postoperative outcome [3,5-7,9-11]. Identifying the associations between preoperative comorbidities, postoperative outcomes, and mortality may contribute to a better understanding of the possible outcomes of patients undergoing esophagectomy and further aid in improving patient care.

The goal of this study is to identify if there are any associations between various patient comorbidities and the risk of developing postoperative complications following partial or full surgical resection of the esophagus.

## Materials and methods

Study design

A historical cohort study was conducted based on a secondary analysis of data. The American College of Surgeons (ACS) National Surgical Quality Improvement Program (NSQIP) database was used to define our patient cohort. A total of 722 hospitals participated in the NSQIP database in 2018. The database collects data on over 150 variables across a 30-day postoperative period with a surgical clinical reviewer capturing the data points via medical chart abstraction. NSQIP undergoes rigorous data collection and audits to ensure all variables have data of high quality. Data are deidentified and, therefore, exempt from approval by Florida International University’s Herbert Wertheim College of Medicine Institutional Review Board.

Sample

Patients older than 18 years were included if they underwent esophagectomy between January 1, 2016, and December 31, 2018, based on Current Procedural Terminology (CPT) codes (43107, 43112, 43119, 43121, and 43122). Patients were excluded if they had disseminated cancer based on clinical pretreatment and/or pathology postsurgery, acute renal failure, infection at the time of surgery, or pneumonia at the time of surgery; were on ventilation, septic, septic shock, or dependent functional status; and had missing data for key variables.

Variables

Independent demographic and patient characteristic variables included in this study were gender, race, age, BMI, weight loss, diabetes mellitus (DM), smoking status, COPD, and congestive heart failure (CHF). Clinical and surgical variables included were the American Society of Anesthesiologists (ASA) physical status classification, esophagectomy type, operation time, length of stay (LOS), and preoperative chemotherapy and/or radiation.

Outcome measures

Postoperative complications included the presence of superficial, deep, or organ/space infection; sepsis; septic shock; pneumonia; unplanned intubations; pulmonary embolism; ventilator use for >48 hours; progressive renal insufficiency; acute renal failure; urinary tract infection; cardiac arrest; myocardial infarction; deep venous thromboembolism; bleeding transfusions; unplanned reoperation; death within 30 days of operation; readmission within 30 days of operation; and anastomotic leak. A composite variable was created to encompass all postoperative complications.

Statistical analyses

Bivariate analyses were conducted to determine the relation between baseline characteristics and potential confounders and the exposure (having a comorbidity), as well as for the association between the exposure and potential confounders and the outcome. Risk-adjusted multivariate logistic regressions were performed to assess the adjusted effect that comorbidities have on rates of postoperative complications. The following variables were used for adjustment: age, gender, race, BMI, diabetes, smoking status, COPD, weight loss, ASA classification, esophagectomy type, and sepsis. Odds ratios (OR) and 95% confidence intervals (CIs) were reported. A *P*-value < 0.05 will be considered significant. All analyses were performed using Stata 16.1 software (StataCorp., College Station, TX).

## Results

A total of 2,698 patients underwent an esophagectomy during 2016-2018. One hundred fifty-four patients did not meet inclusion and exclusion criteria or were missing data. Thus, our study sample comprised 2,544 patients. Table 1 shows the baseline characteristics of the study population. The majority of our population was male (80.8%). The mean age was 63.5 years, with a range of 18 to 90 years. Most of the patients were white (78.2%). About one-third of our patients were classified as overweight (35.4%), followed by obese (29.6%). The minority of our patients were diagnosed with DM (18.5%), COPD (7.9%), CHF (0.5%), or preoperative weight loss (21.4%) or had a smoking history (25.5%). The most frequent surgical procedure was partial esophagectomy, distal two-thirds, with thoracotomy and with/without proximal gastrectomy (52.4%; Table 1), followed by total or near esophagectomy without thoracostomy (21.2%). The median operation time was 350.5 minutes, with an interquartile range of 265 to 454 minutes. The median LOS in the hospital following the procedure was nine days (interquartile range of 7-13 days). Of our sample, 66% received preoperative chemotherapy and/or radiation, with 65.1% receiving preoperative chemotherapy and 56.3% receiving preoperative radiation. Most patients were ASA Class 3 (75.9%) followed by ASA Class 2 (16.9%).

**Table 1:** Demographic and baseline characteristics of patients who underwent esophagectomy from 2016 to 2018 (sample size N = 2,544). *Values are percentages unless indicated. CPT codes: 43107, total or near-total esophagectomy w/o thoracotomy; 43112, total or near-total esophagectomy with thoracotomy; 43117, partial esophagectomy, distal two-thirds, with thoracotomy, and with or w/o proximal gastrectomy; 43121, excision procedures of the esophagus; 43122, partial esophagectomy with or w/o proximal gastrectomy. SD, standard deviation; BMI, body mass index; COPD, chronic obstructive pulmonary disease; CHF, congestive heart failure; CPT, current procedural terminology; IQR, interquartile range; ASA, American Society of Anesthesiologists; NA, not available

Baseline Characteristics	N	%	Range	Missing	%
Gender (Male)	2,055	80.8			
Age (years), mean (SD)	63.5 (10.3)	NA	18-90		
Race, White	1,989	78.2		385	15.13
BMI, mean (SD)	27.1 (5.9)	NA	11-56		
Underweight	118	4.6			
Normal	768	30.2			
Overweight	901	35.4			
Obese	752	29.6			
Diabetes	471	18.5			
Smoking Hx	649	25.5			
COPD	201	7.9			
CHF	12	0.5			
Weight loss	544	21.4			
ASA class				4	0.16
1	8	0.3			
2	431	16.9			
3	1931	75.9			
4	170	6.7			
CPT code					
43107	539	21.2			
43112	373	14.7			
43117	1,332	52.4			
43121	20	0.8			
43122	280	11.0			
Operation time (minutes), median (IQR)	350.5 (265, 454)		15-1,097		
Length of stay, median (IQR)	9 (7, 13)		0-90		
Preoperative chemotherapy	1,656	65.1		27	1.06
Preoperative radiation	1,432	56.3		29	1.14
Preoperative chemo and/or radiation	1679	66.0		27	1.06

The frequency of the 20 postoperative complications following esophagectomy included in our study is presented in Table 2. The most frequent postoperative complications were a return to the operating room (15.7%), anastomotic leak (12.9%), pneumonia (12.7%), bleeding/transfusions (11.8%), readmission (11.4%), and unplanned intubation (10.5%). Each of the remaining 14 complications had an incidence of under 10%.

**Table 2:** Postoperative complication frequency among patients who underwent esophagectomy. DVT, deep vein thrombosis; CPR, cardiopulmonary resuscitation; OR, operating room

Postoperative complications	N	%
Return to OR	399	15.7
Anastomotic leak	327	12.9
Pneumonia	323	12.7
Bleeding transfusions	299	11.8
Readmission	289	11.4
Unplanned intubation	267	10.5
Organ/space infection	240	9.4
On ventilator >48 hours	222	8.7
Septic shock	129	5.1
Sepsis	120	4.7
Superficial infection	112	4.4
DVT/thrombophlebitis	60	2.4
Death within 30 days of operation	58	2.3
Urinary tract infection	51	2.0
Pulmonary embolism	44	1.7
Cardiac arrest requiring CPR	39	1.5
Acute renal failure	26	1.0
Deep incisional infection	24	0.9
Myocardial infarction	22	0.9
Progressive renal failure	15	0.6

The relationship between the baseline characteristics and the presence of one or more postoperative complications is shown in Table 3. Most of the baseline characteristics were shown to have a statistically significant association with the risk of suffering a complication. The only two characteristics that did not have a statistically significant difference between patients with and without complications were weight loss (*P* = 0.257) and esophagectomy type (*P* = 0.133).

**Table 3:** Relationship between baseline characteristics and presence of postoperative complications. BMI, body mass index; COPD, chronic obstructive pulmonary disease

Characteristics	Complication				No complication				*P*-value
Continuous variables	Mean	SD	N	%	Mean	SD	N	%	
Age (years)	64.5	10.2			62.8	10.3			<0.0001
Operation time (minutes)	376.0	129.6			350.3	125.0			<0.0001
Categorical variables									
Gender									0.026
Female			238	48.7			251	51.3	
Male			886	43.1			1,169	56.9	
Race									0.001
White			850	42.7			1,139	57.3	
Not white			95	55.9			75	44.1	
BMI compared to normal									0.001
Underweight	69	58.5			49	41.5			
Normal	342	44.5			426	55.5			
Overweight	363	40.3			538	59.7			
Obese	348	46.3			404	53.7			
Diabetes									<0.001
Yes	242	51.4			229	48.6			
No	882	42.6			1,191	57.5			
Smoking									0.004
Yes	318	49.0			331	51.0			
No	806	42.5			1,089	57.5			
COPD									<0.001
Yes	115	57.2			86	42.8			
No	1009	43.1			1,334	56.9			
Weight loss									0.257
Yes	252	46.3			292	53.7			
No	872	43.6			1,128	56.4			
ASA classification (1 as the reference)									<0.001
1	2	25.0			6	75.0			
2	133	30.9			298	69.1			
3	890	46.1			1,041	53.9			
4	96	56.5			74	43.5			
Esophagectomy type									0.133
Total	421	46.2			491	53.8			
Partial	703	43.1			929	56.9			
Chemotherapy									0.001
Yes	692	41.8			964	58.2			
No	419	48.7			442	51.3			
Radiation									0.005
Yes	597	41.7			835	58.3			
No	513	47.4			570	52.6			
Chemo and/or radiation									
Yes	705	42.0			974	58.0			
No	406	48.4			432	51.6			0.002

Table 4 shows the unadjusted and adjusted ORs for having a postoperative complication according to the baseline and operative characteristics. Adjusted associations were found to be statistically significant for age (OR 1.02, 95% CI 1.01-1.03, and *P* < 0.001), operation time (OR 1.002, 95% CI 1.001-1.003, and *P *< 0.001), race (not white) (OR 1.76, 95% CI 1.26-2.47, and *P* = 0.001), BMI (underweight) (OR 2.18, 95% CI 1.36-3.50, and *P* = 0.001), smoking (OR 1.42, 95% CI 1.14-1.76, and *P* = 0.001), and chemotherapy and/or radiation (OR 0.82, 95% CI 0.68-0.99, and *P* = 0.038).

**Table 4:** Unadjusted and adjusted regressions of the associations between preoperative comorbidities and postoperative complications. The following variables were used for adjustment: age, gender, race, BMI, diabetes, smoking status, COPD, weight loss, ASA classification, esophagectomy type, and sepsis. OR, odds ratio; CI, confidence interval; BMI, body mass index; COPD, chronic obstructive pulmonary disease; ASA, American Society of Anesthesiologists

Characteristics	Unadjusted		Adjusted	
	OR (95% CI)	*P*-value	OR (95% CI)	*P*-value
Age (hours)	1.02 (1.01-1.02)	<0.001	1.02 (1.01-1.03)	<0.001
Operation time (minutes)	1.002 (1.001-1.002)	<0.001	1.002 (1.001-1.003)	<0.001
Gender				
Female	Reference	-	-	-
Male	0.80 (0.66-0.97)	0.001	0.83 (0.66-1.05)	0.117
Race				
White	Reference	-	-	-
Not white	1.70 (1.24-2.33)	0.001	1.76 (1.26-2.47)	0.001
BMI				
Underweight	1.75 (1.18-2.60)	0.005	2.18 (1.36-3.50)	0.001
Normal	Reference	-	-	-
Overweight	0.84 (0.69-1.02)	0.080	0.94 (0.75-1.18)	0.600
Obese	1.07 (0.88-1.31)	0.494	1.20 (0.94-1.54)	0.134
Diabetes	1.43 (1.17-1.74)	0.001	1.23 (0.97-1.55)	0.082
Smoking	1.30 (1.09-1.55)	0.004	1.42 (1.14-1.76)	0.001
COPD	1.77 (1.32-2.37)	<0.001	1.31 (0.94-1.83)	0.111
Weight loss	1.12 (0.92-1.35)	0.257	1.05 (0.84-1.31)	0.680
ASA classification (1 as the reference)				
1	Reference	-	-	-
2	1.34 (0.27-6.72)	0.723	2.41 (0.27-21.64)	0.432
3	2.57 (0.52-12.74)	0.249	4.29 (0.48-38.18)	0.192
4	3.89 (0.76-19.84)	0.102	5.73 (0.63-52.25)	0.122
Esophagectomy type: total	1.13 (0.96-1.33)	0.133	1.16 (0.96-1.39)	0.123
Chemo and/or radiation	0.77 (0.65-0.91)	0.002	0.82 (0.68-0.99)	0.038

## Discussion

This study found numerous preoperative factors to be independently associated with an increased risk of one or more postoperative complications in patients who underwent an esophagectomy during 2016-2018. After adjusted logistic regressions were performed, age, operation time, race (not white), BMI (underweight), and smoking were found to have statistically significant increased odds of experiencing one or more postoperative complications. Patients who underwent chemotherapy and/or radiation preoperatively had statistically significantly decreased odds of postoperative complications. Of the preoperative factors that were found to have an increased risk, underweight BMI had the highest risk of developing one or more complications. The three most common postoperative complications were a return to the operating room, anastomotic leak, and pneumonia; however, we did not separately analyze specific correlations between each preoperative comorbidity and each postoperative outcome in this study.

There are a few published studies that holistically analyze the associations between risk factors and the likelihood of having a complication after esophagectomy. Some studies have evaluated the risks of certain comorbidities and specific complications; however, no studies, to our knowledge, have investigated the risk of developing any postoperative complication with specific risk factors [12,13]. Identifying the associations between preoperative comorbidities and having a postoperative undesirable outcome can aid the physician to educate patients regarding their potential risk of complications and potentially increase the postoperative monitoring for high-risk individuals and lower the associated healthcare costs of complications.

Our initial findings suggested an increased risk of experiencing a postoperative complication in the male sex; however, when an adjusted logistic regression was performed, there was no statistically significant difference in risk between sexes. This is not consistent with other studies that suggested males were at an increased risk for postoperative complications [9]. The general association between the male gender and increased major complication rates may be due to the higher incidence of alcohol consumption and smoking in male patients [14]. Another potential cause of males having increased postoperative complication rates is that cortisol-induced sex hormones vary among sexes, and males are more at risk for complications following surgical stress to the tissue [15]. One study found an association between the female gender and the risk of experiencing a postoperative complication [16]. While our study did not find a significant difference in complication rates between genders, this may be due to our sample size. Based on the current literature, it is important to consider gender during treatment planning, as males may be more susceptible to postoperative complications for a variety of reasons.

In previous studies, obesity has been shown to have protective effects on the occurrence of postoperative complications following esophagectomy (OR 0.78, 95% CI 0.62-0.98, and *P *= 0.02) [10,11]. A theory as to why obesity may have a protective effect is that a higher BMI (25-30) at baseline is associated with more physical reserves of adipose tissue, making the body less prone to protein catabolism [12]. Although obesity is protective, an even higher BMI (>30) has not been considered protective potentially due to the difficulty of surgery with elevated visceral fat compromising operative visibility [12]. Our findings show that neither overweight nor obese BMIs are associated with a significantly increased risk of experiencing complications.

Interestingly, our study found that an underweight BMI was associated with an increased risk of postoperative complications. Low BMI has been reported to be a risk factor for postoperative complications, especially in surgical oncology [17]. Patients with esophageal malignancy can have decreased BMI due to progressive dysphagia, poor appetite and reduced caloric intake due to chemotherapeutic treatment, and/or metastatic disease leading to cachexia [17]. Wightman et al. suggested that patients with low BMI before esophagectomy can have poorer outcomes due to the relation it has with advanced age, skeletal mass loss, and overall frailty, which have all been studied to be risk factors in surgical cases [17]. In their retrospective analysis, Wightman et al. also found that underweight BMI status was significantly associated with an increased risk of pulmonary complications (OR 3.32, 95% CI 2.85-4.12, and *P *= 0.012). They hypothesize that this increased risk can be attributed to impaired nutritional indices, low core muscle mass supporting respiration, and impaired immune function preventing infection. They suggest the possible use of preoperative nutritional supplementation before esophagectomy. Although we did not analyze the specific outcomes associated with underweight BMI, the significant findings in our study can be used to support the optimization of nutritional status with the incorporation of nutritional supplementation.

Although DM has been implicated as a strong predictor of postoperative complications [16], our study along with one conducted by Linden et al. did not find a significant association between DM and experiencing a postoperative complication [18]. DM has been implicated in poor wound healing due to microvascular damage [19,20]. We suspect that our findings are not significant due to the lack of stratification of specific postoperative complications that have been analyzed in previous literature. In their meta-analysis, Li et al. concluded that DM has been significantly associated with the risk of anastomotic leak, a serious complication of esophagectomy due to poor wound healing [19]. Additionally, Okamura et al. analyzed postoperative complications associated with hemoglobin A1c (HbA1c) levels and found significant increases in surgical site infections when HbA1c was greater than 8.0% (*P* = 0.001), anastomotic leaks when HbA1c was greater than 7.0% (*P *< 0.001), and pneumonia when HbA1c was greater than 6.5% [20]. Our findings are limited by the lack of investigation into specific postoperative complications of DM as well as by the lack of information on the study sample about glycemic control.

In their retrospective cohort study, Schlottmann et al. reported an association between Black race and complications following esophagectomy [15]. Our study also found a significant association between race and postoperative complications; however, racial variables were categorically divided into white and nonwhite populations due to low-categorized nonwhite patient populations. Therefore, more specific race associations could not be explored. Our study found that the nonwhite race had a statistically significant risk of developing a postoperative complication as compared to the white race only. Both Erhunmwunsee et al. and Chen et al. found links between complication rate and socioeconomic status, citing this as an explanation for race being associated with postoperative complication rate, not the race itself [13,21]. Another study investigating mortality in patients undergoing esophagectomy found that there was a reduced survival time among those with lower socioeconomic status who underwent esophagectomy [13,22]. Our study did not analyze socioeconomic status due to the lack of the necessary data in the source database.

One study found increasing age to be a predictor of postoperative complications [19]. Our study found similar results. The literature points to a variety of reasons why increased age may be a predictor of postoperative outcomes. Such theories include the decline of physiological reserve function, accompanying acute and chronic disease and potential malnutrition, lower immune function, and anemia [20]. Clinically, increasing age should be taken into consideration during treatment planning alongside other patient factors to ensure the safest treatment for the individual patient. Given the average age of individuals who undergo esophagectomy is 58.7 years, our study may support consideration for other treatment modalities of esophageal malignancy, such as chemotherapy and/or radiation, given the risk with increasing age [23].

Smoking tobacco has been implicated as a general risk factor for developing malignancies, as well as a risk factor in wound healing [24,25]. This is due to smoking leading to inflammation and dysregulation of inflammatory and protective mediators. These effects due to smoking have been shown to leave the patient more susceptible to poor long-term survival, the development of acute respiratory distress syndrome, and inflammatory alveolar edema [26]. Our study supported this well-known association between smoking and surgical complications; however, we did not investigate which complication it is most closely associated with in this analysis.

Studies that evaluate the association between neoadjuvant chemotherapy and/or radiation and postoperative complications have produced conflicting results. Many factors may influence the outcomes of patients who undergo neoadjuvant therapy, such as disease progression and the timing of the procedure following therapy [27]. In our study, chemotherapy and/or radiation significantly decreased the odds of a postoperative complication following esophagectomy (OR 0.82, 95% CI 0.68-0.99, and *P* = 0.38). Although studies by Yang et al. and Cunningham et al. showed that neoadjuvant therapy improved the long-term survival of patients with esophageal malignancies, other studies showed no statistically significant protective effect on the rates of complications other than mortality [27-28]. The effect of neoadjuvant therapy on postoperative complications following esophagectomy should be further investigated as this is an important vehicle in the management of oncologic conditions.

Finally, it is important to note that mortality was not included as an outcome in our study. It was ultimately excluded due to a low mortality rate among our study sample, which we initially attributed to the short-term follow-up period (30 days after surgery). A previous study described 90-day mortality to be more than double the 30-day mortality rate (8.9% versus 4.2%; *P* < 0.0001) [29]. The study reported that mortality in the first 30 days was influenced most by age and comorbidities. This is compared to 90-day mortality which was most influenced by characteristics of the patient’s malignancy such as stage and location. This suggests that 30-day postoperative mortality may be more informative and targeted to the effect of comorbidities on patient outcomes compared to the 90-day follow-up.

Strengths and limitations

A strength of this study is the use of the NSQIP database, as it is a nationally validated database that is specifically designed for surgical outcomes and quality control. This allows for objective data to be analyzed providing reliable data points and a large sample size. NSQIP also allows many risk factors and outcomes to be assessed at one time, thus making this study more clinically relevant.

While NSQIP is a robust database, it also limits our study based on its dictated terms. The short follow-up time of 30 days may have led to an underestimation of complication incidences and the overall mortality rate. Additionally, hospitals voluntarily choose to be a part of the NSQIP, which may impact the generalizability of our study to patients who undergo esophagectomy. NSQIP also does not have complete data on tumor staging; therefore, it may not be known if a patient has metastatic disease, thereby increasing the risk of postoperative complications. Although we had a robust sample size, having a larger sample size would have added strength to our study by improving our study’s CIs.

Another limitation of our study was that we did not analyze the associations between individual comorbidities and postoperative complications, which should be examined in future studies. Finally, the severity of the individual risk factors could not be measured on a severity scale but was instead identified to be present or not present. Although it may be useful to note that the presence or absence of a risk factor may be useful in directing treatment or explaining possible outcomes, clinically, the severity of the risk factor may be more useful in suggesting the risk of development of postoperative outcomes. In addition, various residual confounding variables may not have been accounted for in this study which may have biased our results.

Future directions

Investigation into individual comorbidities and their associated postoperative complications is planned for future research with this preliminary data. By understanding which comorbidities can lead to each postoperative event, patients can be triaged accordingly to decrease complications and mortalities. As an anastomotic leak is one of the most feared complications, in future studies, it would be beneficial to investigate the specific comorbidities that increase the risk of an anastomotic leak following esophagectomy. More specifically, it is imperative to investigate the associations between DM and anastomotic leak due to the condition’s association with poor wound healing. Additionally, although operation time was found to be significant in this study, we did not further investigate this association due to the degree of significance and suggest future studies evaluate this parameter. Finally, with our results showing chemotherapy and/or radiation therapy serving as a protective measure, the literature describes neoadjuvant therapy conflicting results in the development of postoperative complications following esophagectomy. Due to this disagreement, we encourage future investigation into the risk of experiencing complications following neoadjuvant therapy.

## Conclusions

This study is one of the first to assess the association between a specific preoperative risk factor and developing any of the predefined postoperative complications following esophagectomy, the gold standard treatment for esophageal malignancy. Our study found that age, operation time, race (not white), BMI (underweight), and smoking were independently associated with an increased risk of developing a postoperative complication. Our results also showed that neoadjuvant chemotherapy and chemotherapy and/or radiation served as protective measures in the development of complications.
